# Sequential simulation (SqS) of clinical pathways: a tool for public and patient engagement in point-of-care diagnostics

**DOI:** 10.1136/bmjopen-2016-011043

**Published:** 2016-09-13

**Authors:** Jeremy R Huddy, Sharon-Marie Weldon, Shvaita Ralhan, Tim Painter, George B Hanna, Roger Kneebone, Fernando Bello

**Affiliations:** 1 Department of Surgery and Cancer, Imperial College, London, UK; 2 Patient Representative, London Cancer Alliance, London, UK

**Keywords:** Point-of-Care Testing, Patient Engagement, Patient Simulation, Volatile Organic Compounds

## Abstract

**Objectives:**

Public and patient engagement (PPE) is fundamental to healthcare research. To facilitate effective engagement in novel point-of-care tests (POCTs), the test and downstream consequences of the result need to be considered. Sequential simulation (SqS) is a tool to represent patient journeys and the effects of intervention at each and subsequent stages. This case study presents a process evaluation of SqS as a tool for PPE in the development of a volatile organic compound-based breath test POCT for the diagnosis of oesophagogastric (OG) cancer.

**Setting:**

Three 3-hour workshops in central London.

**Participants:**

38 members of public attended a workshop, 26 (68%) had no prior experience of the OG cancer diagnostic pathway.

**Interventions:**

Clinical pathway SqS was developed from a storyboard of a patient, played by an actor, noticing symptoms of oesophageal cancer and following a typical diagnostic pathway. The proposed breath testing strategy was then introduced and incorporated into a second SqS to demonstrate pathway impact. Facilitated group discussions followed each SqS.

**Primary and secondary outcome measures:**

Evaluation was conducted through pre-event and postevent questionnaires, field notes and analysis of audiovisual recordings.

**Results:**

38 participants attended a workshop. All participants agreed they were able to contribute to discussions and like the idea of an OG cancer breath test. Five themes emerged related to the proposed new breath test including awareness of OG cancer, barriers to testing and diagnosis, design of new test device, new clinical pathway and placement of test device. 3 themes emerged related to the use of SqS: participatory engagement, simulation and empathetic engagement, and why participants attended.

**Conclusions:**

SqS facilitated a shared immersive experience for participants and researchers that led to the coconstruction of knowledge that will guide future research activities and be of value to stakeholders concerned with the invention and adoption of POCT.

**Strengths and limitations of this study**

- This article presents the use of sequential simulation (SqS) as a tool for public and patient engagement in new point-of-care diagnostic testing strategies.
- SqS allows participants to appreciate the patient journey, including the possible downstream pathway consequences of introducing new diagnostic strategies.
- This shared immersion of patients, publics and researchers allows coconstruction of knowledge that will guide future research, evidence generation and policy.
- Patient engagement necessitates voluntary participation and convenience sampling was unavoidable.
- The test device under evaluation is at a relatively early stage of development and therefore the SqS scenarios were based on assumptions.

## Background

Public and patient engagement (PPE) is a fundamental component of healthcare research and actively encouraged by the Department of Health and major funding bodies. PPE refers to the dissemination of information and knowledge between healthcare providers and researchers, members of the public (including patients, carers and people who use health and social care services) and members of organisations representing service users.^1^ This participation ensures research is applicable to patients, and therefore, increases the likelihood that research findings will translate into clinical practice^2^ as well as addressing the ethical and political requirements of PPE within healthcare research.^3^ ^4^ Involving patients in the management of their healthcare is widely recognised to improve quality of care, patient safety and health outcomes.^5–8^ However, PPE can be expensive and, if not rigorously undertaken with appropriate methodologies, there is a risk of reducing it to a mechanistic ‘paper exercise’ that restricts the quality of information generated from the process.^4^ Ocloo *et al*^9^ in their recent narrative review of patient and public involvement (PPI) in healthcare call for ‘models and frameworks that enable power and decision-making to be shared more equitably with patients and the public in designing, planning and co-producing healthcare’.

PPE is particularly important in the implementation of innovation and healthcare delivery improvements.^10^ Technology advancement such as new diagnostics play an important role in such innovation but development must be in keeping with patient's health needs and expectations.^11^ ^12^ More tests are making the translation from the laboratory to point-of-care test (POCT) devices, creating new diagnostic strategies that may disrupt traditional clinical pathways. It is rare for a patient to directly benefit from a diagnostic test in isolation;^13^ instead the impact comes from the decisions and interventions that are undertaken as a result of its introduction. Therefore, to allow informed input from public and patients into the research and development of novel diagnostic tests, the test process and any subsequent effects to downstream clinical pathways must be demonstrated.

Sequential simulation (SqS) is a tool that demonstrates key elements in a patient's journey and can highlight the consequences of intervention at each step. SqS workshops can provide an innovative framework for PPE, especially when aligned to the four key assumptions of the Shared Immersion Model as described by Tang *et al*:^14^

- Public engagement activities can be experiential, involving participation as well as acquisition of information
- The shared experience constitutes an event
- Being immersed in the shared experience is central, for researchers and publics alike. Critical evaluation of this process is equally valuable for both
- Simulation can provide immersive engagements between science and publics, especially with healthcare activity and research

Simulation has previously been used by our group as a tool for engagement with integrated care,^15^ multidisciplinary teams and adolescents^16^ and for surgical devices.^17^ In the first two examples, SqS addressed clinical care pathway redesign; while in the latter, the focus was on the use of novel devices in healthcare environments. The current study combines these two approaches to facilitate PPE in the development of POCT diagnostics by exploring the stakeholder perspective on a new diagnostic test device and investigating its potential impact on the patient's journey.

The aim of this study is to introduce, through a case study, SqS as a methodology for active PPE in the development of novel POCTs and appraise the approach.

## Case study

Oesophagogastric (OG) cancer represents the fourth and fifth most common types of cancer death.^11^ Each year in England, 12 900 people are diagnosed with OG cancer and of these only 37.3% are considered curable at the time of diagnosis.^11^ ^12^ However, when diagnosed at its earliest stage, the 1-year survival is 75–87%.^11^ Public awareness of these cancer types is poor and the symptoms (dyspepsia (heartburn or indigestion), dysphagia (difficulty swallowing), unexplained weight loss, persistent vomiting, tiredness (anaemia) and upper abdominal pain) are common and often not associated with cancer.^10^ This leads to a delay in presentation to healthcare services and therefore treatment. In view of this, National Health Service (NHS) England recently undertook a ‘Be Clear on Cancer’ campaign for OG cancer targeting patients with heartburn and indigestion.^18^ Within our department, research is investigating the use of volatile organic compounds (VOCs) for early OG cancer diagnosis. Previous experimental work has demonstrated that exhaled breath analysis using mass spectrometry can distinguish oesophageal and gastric adenocarcinoma from non-cancer controls,^19^ demonstrating the potential for a VOC-based breath test to provide point-of-care risk stratification for patients with suspected OG cancer. This new approach aims to provide a readily available, non-invasive and cost-effective test to streamline patients with the early symptoms of cancer to further investigation with endoscopy. It is hoped that by increasing the number of patients diagnosed early, a higher proportion of these patients will have early stage disease and be able to enter a treatment pathway with curative intent. Much remains to be learnt about the social, cultural and practical implications of this new technology.

## Methods

Three 3-hour workshops were undertaken in central London. For workshop 1, we invited members of the Oesophageal Patients Association through their mailing list and social media, providing access to participants with prior experience of OG cancer and its diagnostic pathway, either personally or as a relative or carer. Workshops 2 and 3 were aimed at the general public without previous experience of OG cancer. Recruitment to workshop 2 was through an open invitation advertised in venues local to the workshop, and in workshop 3 a focus group recruitment firm (Focus4People, Herts, UK) was used.

**Figure 1:**
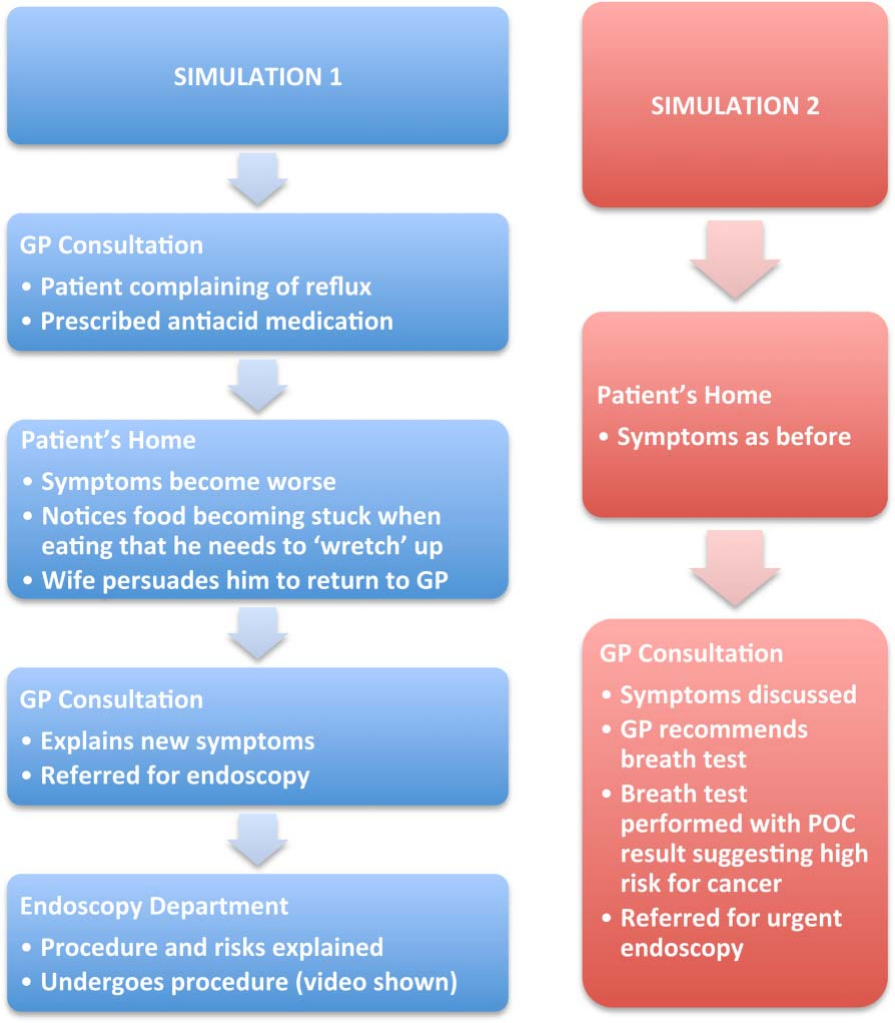
Storyboard of diagnostic pathway for oesophagogastric cancer used in sequential simulation scenarios. GP, general practitioner; POC, point-of-care.

**Figure 2:**
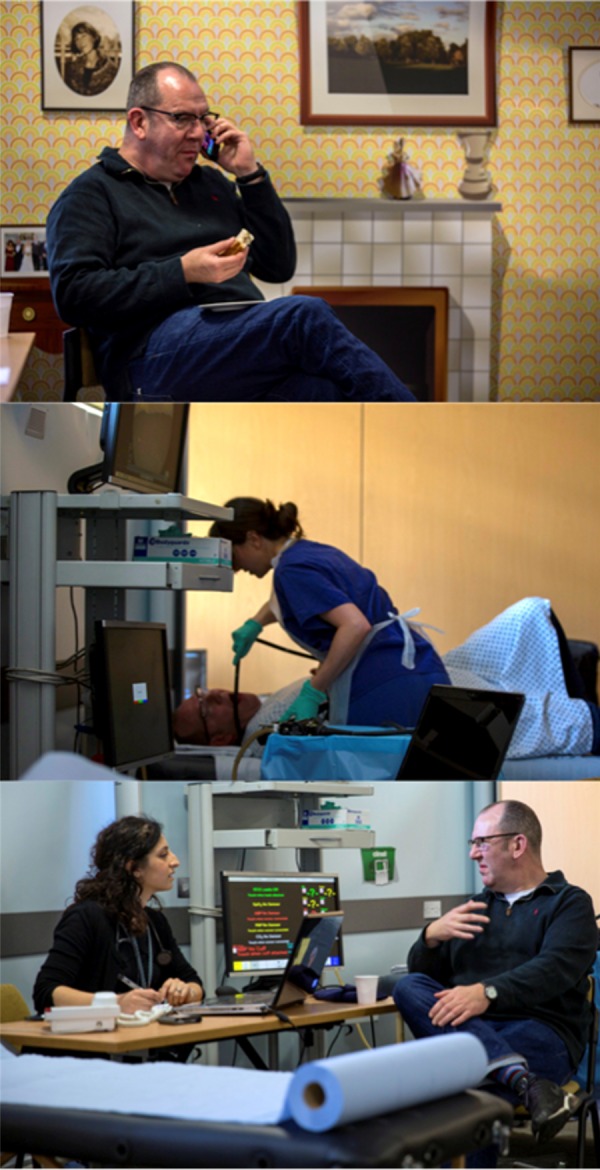
Sequential simulation (SqS) sets to illustrate (i) the patient's home (ii) an endoscopy unit and (iii) general practice consultation.

The three workshops followed the same structure and comprised presentations, clinical pathway SqS and facilitated group discussions. Following a brief introduction to the day, a presentation covering the background to OG cancer including the current diagnostic strategy was given. This led into the first clinical pathway simulation—a scenario that was designed to represent a relatively common patient journey. It was unscripted and followed a storyboard of a patient, played by a professional actor, noticing the symptoms of an oesophageal cancer and undergoing a typical diagnostic pathway (figure 1). The storyboards were designed to represent a ‘typical’ uncomplicated diagnostic pathway for a patient with oesophageal cancer and created in consultation with specialist clinicians. To validate the storyboard participants with a personal experience of OG cancer, were asked to feedback on how the scenario related to their own experiences during the first workshop. Actors were used to ensure a personalised approach to the scenario, while maintaining confidentiality and ensuring consistency across workshops. Clinicians played their own healthcare roles in the simulation scenarios. The clinicians also respond well to actors who appear very realistic. The sets were created using distributed simulation^20^—versatile transportable screens and props including beds, desks and medical equipment (figure 2). The simulation scenario was followed by the workshop breaking down into small groups (5–10 people) for facilitated table discussions regarding the current diagnostic pathway. The groups were then brought back together and table discussions were summarised in a plenary session. After a coffee break, a member of the research team gave a presentation describing the current research achievements in lay terms and introduced the concept of the novel breath test, as well as our future ambitions. A second SqS followed the same patient through his journey, although this time we incorporated the breath test into the scenario to demonstrate the potential downstream consequences to the clinical pathway. Again, small table discussions followed.

Participants completed pre-event (see online supplementary appendix 1) and postevent (see online supplementary appendix 2) questionnaires that included a series of statements regarding attitudes and feedback related to the novel test device and also on the SqS workshop experience itself that participants were asked to rate on a 5-point Likert scale (strongly disagree to strongly agree). Participants were given copies of presentation slides and contact details if they would like to discuss anything from the workshop further.

10.1136/gutjnl-2015-311146.supp1 Supplementary appendix

10.1136/gutjnl-2015-311146.supp2 Supplementary appendix

The workshop discussions were filmed and recorded for subsequent analysis. Workshop recordings were transcribed and emergent thematic analysis undertaken independently by two members of the research team (JRH and SR), who were not directly involved in the test device research. Qualitative data were analysed with NVivo V.10.1.1 software (QSR International, Melbourne, Australia). Ethical approval for the workshops was provided by the Imperial College Joint Research Compliance Office (reference number ICREC_11_5_8) and informed consent was obtained from all workshop participants.

## Results

In total, 38 participants attended the 3 workshops, 26 (68%) of whom had no previous experience personally or as a relative or carer of the OG cancer diagnostic pathway. A total of 12 participants were recruited through the Oesophageal Patients Association, of which 11 (92%) attended; 14 participants replied to public poster and email campaign, of which 8 (57%) attended; and 20 patients were recruited by the focus group recruitment company, of which 19 (95%) attended. Two participants have subsequently volunteered to contribute further to our research activities and now have a role in PPI within the group.

### Thematic analysis of workshop audio recordings

Two analyses were undertaken: (i) an analysis of participant attitudes towards the use of SqS for PPE in POCT diagnostics; and (ii) an analysis of patient feedback regarding the breath test device, diagnostic strategy and clinical pathway.

Three themes emerged regarding the use of SqS for public engagement in novel POCT diagnostic test: participatory engagement, simulation and empathetic engagement, and why participants attended. Theme summaries and quotations from transcripts are included in table 1.

**Table 1:** Summary of themes relating to use of sequential simulation for public engagement in novel diagnostics

Key themes identified	Summary	Quotations from transcript
Participatory engagement	All participants actively contributed to small group discussions that often overran and continued into breaks and over lunch.	‘We were made comfortable to contribute’. W2 ‘Feeling that the patient perspective is important in achieving better results’. W2
Simulation and empathetic engagement	Participants felt the simulations gave structure to the events allowing them to focus on the simulated patients’ journey and understand the consequences of the new diagnostic strategies. Participants who had been through the diagnostic pathway of OG cancer commented how similar it was to their experiences although there were some comments that the pathway was oversimplified in some areas, for example, ease of access to GP appointment and ease in which the GP undertook the test within a consultation.	‘The process I just watched echoed exactly what I experienced when I was diagnosed’. W1 ‘The simulation of patient/GP scenarios to add context’. W2 ‘It really stays in your mind, you know, you have a picture’. W2 ‘It makes it more real’. W2
Why participants attended	Attendance at the workshops was linked to the method of recruitment. In the second workshop that was recruited through advertisements in the local areas (see methods), we explored the motivation for attendance. This was broadly divided into those with a community interest in new diagnostic and medical developments and those that had experience of cancer either personally or as family or carers and wanted to learn more and contribute to research in this area.	‘I am interested in this way of, it's not only researchers and doctors thinking about how to develop it, but they are also listening carefully to how people would feel with these new developments’. W2 ‘This (workshop) interested me because as it just so happens last year I had three gastroscopies and I know what they feel like’ W2 ‘I organize health workshops with community development, with community organisations, we work in the local community and we stress prevention and that sort of thing’. W2

W1, W2 or W3 denotes workshop where quotation was made.

GP, general practitioner; OG, oesophagogastric; W, workshop.

In respect to the diagnosis of OG cancer and the proposed new breath test and diagnostic test strategy, five themes emerged from the workshop: awareness of OG cancer, barriers to testing and diagnosis, design of new test device, new clinical pathway and placement of test device. Theme summaries and participant quotations are presented in table 2.

**Table 2:** Summary of themes relating to current and novel diagnostic strategy for Oesophago-gastric cancer

Key themes identified	Summary	Quotations from transcript
Awareness of oesophagogastric cancer	There was general agreement regarding the lack of awareness of OG cancer, it's symptoms and the poor outcome. There was a strong feeling of a need for awareness campaigns including celebrity endorsement and social media. The role of the media in raising awareness was discussed, and it was felt that over the counter antacid medications should contain warnings of the disease.	‘It needs to be more like colorectal cancer because people know if they have blood in their stools it's a worrying sign and straight away they go to their GP’. W1 ‘Media really important’! W2 ‘GPs see this condition rarely compared with other cancers’. W1 ‘If you are buying so and so amounts of Gaviscon this could be a sign (of cancer)…please check with your GP’. W3 ‘I didn't realise there was such a cancer at such a low survival rate’. W3
Barriers to testing and diagnosis	There were many barriers highlighted to the current diagnosis of OG cancer; these included the anxiety, invasiveness, cost and complications of endoscopy, lack of education, cultural reluctance to seeking medical advice particularly among men in at risk age groups, difficulty in accessing primary care services, delayed recognition of potential cancer and subsequent referral by GPs and hospital delays for investigation.	‘Males more difficult to get to present and further awareness needed and less-invasive options’. W2 ‘How invasive the current test is could put a lot of people off’. W1
Design of new test device	A potential urine test and breath test were acceptable, but overall most participants would prefer a breath test. There was a preference for a compact box like test similar to a police breathalyser (offers familiarity). Participants would rather see the test launched early and not delayed for attempts to improve accuracy. How the test provided results was a controversial topic with some participant preferring a quantitative result, for example, a risk percentage, some would prefer to have triage-like results, for example, high risk or low risk. A pure binary result, for example, red light/green light was not popular as it was felt this would increase anxiety. What overrides these discussions was that the practitioner delivering the result should be appropriately trained to explain the meaning of the result and council regarding further management and privacy and support need to be provided. Written information was not felt to be sufficient and the inclusion of a nurse was highlighted as beneficial.	‘It is important that people are trained to give out the result appropriately’. W1 ‘The simpler, the better’. W1 ‘Person giving results has to be able to quantify the risk (eg, percentage chance) of cancer when they give the test result—“high-risk” is too vague’. W1 ‘It is important that people are trained to give out the results appropriately’. W1 ‘I actually liked the box with a mouthpiece rather than a plastic bag’. W3 ‘I don't mind what kind of test as long as it's accurate’. W3 ‘Needs to be brought to market faster’
New clinical pathway	There was an overall positive response to the proposed new pathway incorporating the potential breath test device. Participants felt that increasing access and convenience to diagnosis would encourage uptake, particularly in a non-invasive test device. There was some concern that a positive breath test would increase anxiety preceding endoscopy, but this was felt to be unavoidable and would always occur in cancer diagnostic pathways and may in fact increase the uptake of subsequent endoscopy. It was important that patients with a negative breath test know to return to their GP, if symptoms do not improve.	‘Something before the invasive endoscopy test would encourage people more’. W1 ‘If there was a recurring acid problem and the breath test was negative I would probably want to go for an endoscopy’. W3 ‘The anxiety has to come at a point so maybe he just has to go through it earlier’. W1
Placement of novel test device	Most participants felt the test should be placed in either a *GP surgery* or a *pharmacy*. Other proposed testing locations were: Public places for ‘drop-in’ testing Workplace testing Health fairs Mobile testing to ‘at-risk’ populations Booths in GP practices Home testing	‘If there is an easy test maybe it should be available in a more accessible place than the GP practice’. W1 ‘If the test is to be used in a pharmacy perhaps just having high risk or low risk is sufficient, or perhaps just that you need to have more tests or you don't’. W1 ‘Are we going to have the approach like the radiographers that do imaging and then say they can't interpret the results and you will go to your (doctor's) appointment’. W1

W1, W2 or W3 denotes workshop where quotation was made.

GP, general practitioner; OG, oesophagogastric; W, workshop.

Table 3 demonstrates the ‘co-construction of knowledge’ document. This collective output of shared experiences from researchers, patients and public summarises the key outputs raised from the workshops with proposed solutions to guide future research and implementation strategies.

**Table 3:** Coconstruction of knowledge from researcher–participant shared experience

Issues raised	Solutions
Poor awareness of OG cancer	Better media campaigns (a media campaign was initiated by NHS England during the course of this study), involvement of celebrities and social media Highlighting symptoms of OG cancer on antacid medication packets akin to health warnings on cigarette packaging
Invasiveness and risks of endoscopy deterring patients presenting to medical services	Breath test could triage for endoscopy Improve cost-effectiveness of patient journey
Breath test device design	Preference for compact (box like) simplistic breath test over urine Significant number of people not concerned about aesthetic appearance Preference for breath test over urine
Positioning of test: pharmacy versus GP	Preference for pharmacy for ease of access and speed in which test can be done Concerns regarding meaning of test result and how it will be explained to patients Hybrid options such as test being undertaken in pharmacy and result explained by GP/hospital doctor at a later date
Delivery of test results and impact on patients	Professional delivery of breath test results including quantification, explanation of future expectation will require, excellent communication skills and relevant knowledge Training for all involved will be essential GPs likely to have optimum skill set to achieve this

GP, general practitioner; OG, oesophagogastric; NHS, National Health Service.

### Participant feedback questionnaire

All patients completed a questionnaire at the start and on completion of the workshop.

**Figure 3:**
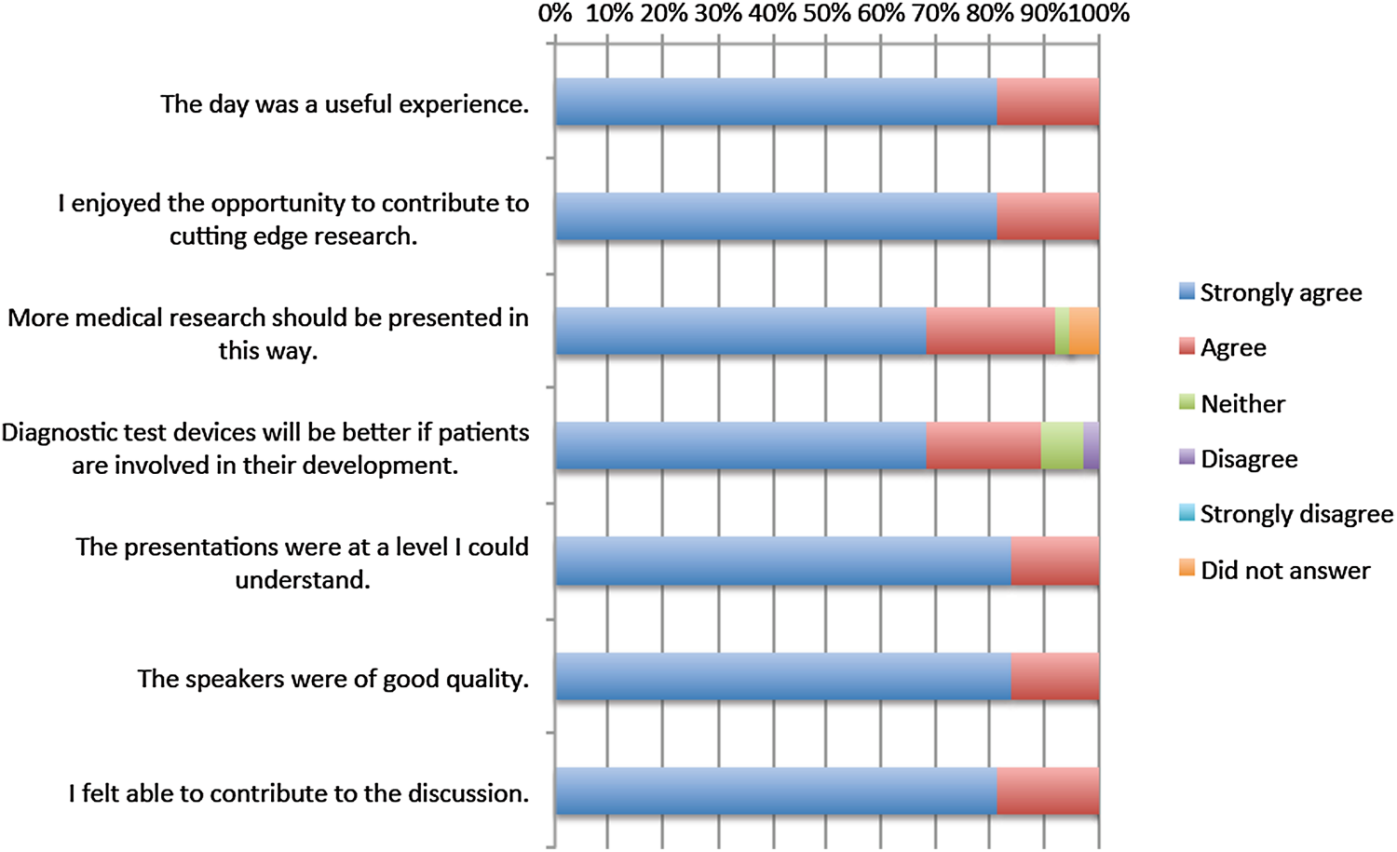
Participant questionnaire feedback relating to the sequential simulation (SqS) workshop experience.

Feedback relating to the SqS workshop experience is shown in figure 3. All patients reported they felt able to contribute to the discussion (strongly agree n=31 (82%); agree n=7 (18%)), that presentations were at a level they were able to understand (strongly agree n=32 (84%); agree n=6 (16%)) and that the day was a useful experience (strongly agree n=31 (82%); n=7 (18%)). The majority of participants felt that diagnostic test devices will be better if patients are involved in their development (strongly agree n=26 (68%); agree n=8 (21%); neither agree or disagree n=3 (8%); disagree n=1 (3%)).

**Figure 4:**
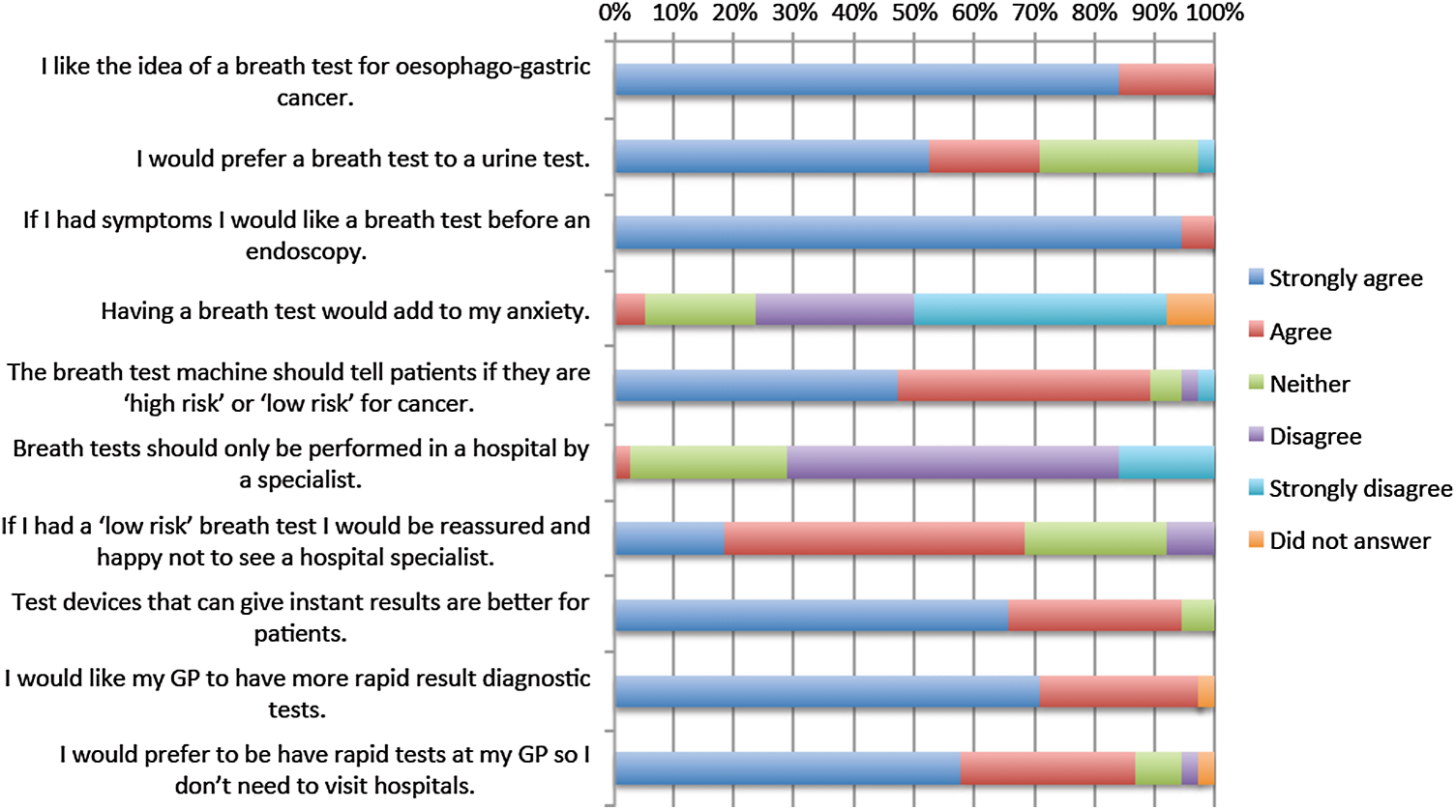
Participant questionnaire feedback relating to the novel breath testing strategy for the diagnosis of oesophagogastric cancer. GP, general practitioner.

Feedback relating to the breath test strategy is shown in figure 4. All patients liked the idea of a breath test for OG cancer (strongly agree n=32 (84%); agree n=6 (16%)) and stated they would like a breath test before an endoscopy (strongly agree n=36 (95%); agree n=2 (5%)). The majority of patients did not agree that the breath test would add to their anxiety (agree n=2 (5%); neither agree or disagree n=7 (18%); disagree n=10 (26%); strongly disagree n=16 (42%); 3 (8%) did not answer) and agreed that a ‘low-risk’ result from the breath test would provide reassurance without specialist referral (strongly agree n=7 (18%); agree n=19 (50%); neither agree or disagree n=9 (24%); disagree n=3 (8%)).

## Discussion

SqS workshops provided an effective immersive environment for public and patient engagement around device development and future diagnostic strategy, creating a shared experience that was beneficial to participants and researchers. Rich data provide feedback on research activities to date and will help guide future research activities.

The outputs from the current workshops have already influenced future research plans regarding the VOCs breath test for OG cancer as follows: identifying a broader scope of where the test could be located, including general practice, pharmacies and workplaces; how the test result is presented to manage associated patient anxiety; and the migration from currently used breath bags to sensor technology. The workshops confirmed patient acceptability of the proposed breath testing strategy for cancer diagnosis and reinforced the lack of awareness that exists with regard to OG cancer and its symptoms.^21^ Furthermore, the workshop outputs have provided valuable information to other stakeholders concerned with POCT invention and adoption, including device designers, clinicians, commissioners and policymakers. As such, we believe results from PPE activities should be routinely published alongside clinical studies and cost-effectiveness arguments as part of a complete evidence package.

The largest challenge in running the workshops was recruitment. Three strategies were trialled, providing an opportunity to weigh the benefits and challenges of each and to recruit different participants with different backgrounds and motivations. The professional focus group recruitment firm was convenient and allowed the tailoring of the group to ensure a representative sample. However, this entailed greater cost as a recruitment commission was paid to the company. Recruitment to workshop 1 was also straightforward as replies to invitations sent to members of the Oesophageal Patients Association were forthcoming. Workshop 2 provided the greatest challenge in recruitment, requiring members of the research group to explore the locality of the workshop venue and placing posters in a variety of venues to encourage interest. Response rate was low, and there was a high rate of non-attendance on the day.

Other costs associated with running the workshops were venue hire including refreshments and lunch, professional actor fees, scenery and props for the simulation scenarios, and transport of equipment. PPI workshops do have an associated cost, for these three events we allocated a budget of £12 000 (although this included the purchase of equipment that can be reused in subsequent events); it is therefore vital that the cost of PPE activities be incorporated into research budgets and funding applications.

This methodology does have limitations. Patient engagement necessitates voluntary participation, and convenience sampling was unavoidable. By varying the recruitment strategy, incentives for attendance and previous knowledge of OG cancer, we hope to have achieved an overall representative sample in the course of the three events, although sociocultural and clinical characteristics of participants were not recorded. Screening participants for their level of engagement using the Patient Health Engagement Scale^22^ or Patient Activation Measure^6^ would have provided a useful measure to interpret the comments of participants. The transcripts from the events were analysed by two researchers who were not directly involved with the development of the breath test or VOC research, minimising bias in emergent theme analysis. Finally, the test device under evaluation is at a relatively early stage of development and therefore the scenarios were based on assumptions.

Patient engagement is vital at this early stage to guide device development, ensure the proposed clinical pathway is acceptable to patients and guide future research activities. To be effective, patient engagement must not be seen as an isolated event and needs to continue alongside evidence generation and device development to improve the acceptability of POCT.

The value of PPE is increasingly recognised in the development of medical devices and care pathways. Barello *et al*,^7^ in their systematic review of eHealth for Patient Engagement, describe PPE as multidimensional with behavioural, cognitive and emotional components. The review concludes that the majority of approaches to PPE do not facilitate engagement at all three levels. However, SqS is able to provide a holistic and systematic approach that fulfils all three as described in the shared immersion model^14^ through the participant's role in the experience (emotional), evaluation (cognitive) and participation in the ongoing process of device development (behavioural). Furthermore, the nature of the shared event allows a degree of empathy between all stakeholders present, so that the outputs from the workshops can truly be underpinned in further research and device development.

In conclusion, SqS provides an effective methodology for active public and patient engagement and, to a limited degree, involvement in research towards the development of new POCT diagnostic devices and testing strategies. The outputs of these events provide rich data that can be of use to a wide range of stakeholders and could form a routine part of the evidence base, informing the adoption of new POCT devices.
