# Supplementary material for: Sequential simulation (SqS) of clinical pathways: a tool for public and patient engagement in point-of-care diagnostics
Source: BMJ Open. 2016 Sep 13;6(9):e011043. doi: 10.1136/bmjopen-2016-011043 (PMC5030544; doi:10.1136/bmjopen-2016-011043)
Supplement: Supplementary appendix [file bmjopen-2016-011043supp_appendix1.pdf]

Appendix 1 Pre-event Questionnaire

Pre-Event Feedback Form

We would like to thank you for attendance and participation today. We would like to gage your understanding of issues described in today's events before the day commences.

When you complete the questionnaire please pass it to your facilitator at the end of the day.

**I am a:**	Patient	Relative of Patient	Clinician	Other
	☐	☐	☐<br>Role:	☐<br>Role:

In regards the diagnosis of Oesophago-gastric Cancer:

	Strongly Agree	Agree	Neither agree and disagree	Disagree	Strongly Disagree
I have a clear understanding of the care pathway for Oesophago-gastric Cancer	☐	☐	☐	☐	☐
I have personal or family experience of Oesophago-gastric Cancer	☐	☐	☐	☐	☐
The current system can cause anxiety	☐	☐	☐	☐	☐
Test devices that can give instant results are better for patients.	☐	☐	☐	☐	☐
I would like my GP to have more rapid result diagnostic tests.	☐	☐	☐	☐	☐
I would prefer to be have rapid tests at my GP so I don't need to visit hospitals.	☐	☐	☐	☐	☐

How much do you know about the term Sequential Simulation (SqS)?

No idea ☐ Some idea ☐ A lot ☐

Comments:

____

____

____

____

______

How much do you know about how different parts of healthcare systems link up?

Nothing ☐ A little bit ☐ A lot ☐

Comments:

____

____

____

____

______

My understanding of today's event is…

Thank you for your feedback.
