# Supplementary material for: Sequential simulation (SqS) of clinical pathways: a tool for public and patient engagement in point-of-care diagnostics
Source: BMJ Open. 2016 Sep 13;6(9):e011043. doi: 10.1136/bmjopen-2016-011043 (PMC5030544; doi:10.1136/bmjopen-2016-011043)
Supplement: Supplementary appendix [file bmjopen-2016-011043supp_appendix2.pdf]

Appendix 2 Post-event Questionaire

Post-Event Feedback Form

We would like to thank you for attendance and participation today. We welcome your comments about today's event.

When you complete the questionnaire please pass it to your facilitator at the end of the day.

**I am a:**	Patient	Relative of Patient	Clinician	Other
	☐	☐	☐<br>Role:	☐<br>Role:

In regards the diagnosis of Oesophago-gastric Cancer:

	Strongly Agree	Agree	Neither agree and disagree	Disagree	Strongly Disagree
I like the idea of a breath test for oesophago-gastric cancer.	☐	☐	☐	☐	☐
I would prefer a breath test to a urine test.	☐	☐	☐	☐	☐
If I had symptoms I would like a breath test before an endoscopy.	☐	☐	☐	☐	☐
Having a breath test would add to my anxiety.	☐	☐	☐	☐	☐
The breath test machine should tell patients if they are 'high risk' or 'low risk' for cancer.	☐	☐	☐	☐	☐
Breath tests should only be performed in a hospital by a specialist.	☐	☐	☐	☐	☐
If I had a 'low risk' breath test I would be reassured and happy not to see a hospital specialist.	☐	☐	☐	☐	☐
Test devices that can give instant results are better for patients.	☐	☐	☐	☐	☐
I would like my GP to have more rapid result diagnostic tests.	☐	☐	☐	☐	☐
I would prefer to be have rapid tests at my GP so I don't need to visit hospitals.	☐	☐	☐	☐	☐

In regards to today's event:

	Strongly Agree	Agree	Neither agree and disagree	Disagree	Strongly Disagree
The day was a useful experience.	☐	☐	☐	☐	☐
I enjoyed the opportunity to contribute to cutting edge research.	☐	☐	☐	☐	☐
More medical research should be presented in this way.	☐	☐	☐	☐	☐
Diagnostic test devices will be better if patients are involved in their development.	☐	☐	☐	☐	☐

The presentations were at a level I could understand.	☐	☐	☐	☐	☐
The speakers were of good quality.	☐	☐	☐	☐	☐
I felt able to contribute to the discussion.	☐	☐	☐	☐	☐

Other questions:

One thing I enjoyed most about today:
One thing I would change about today:
My thoughts on the breath test:

Thank you for your feedback.
